# A linear-nonlinear model accurately predicts cortical responses to simultaneous electrical stimulation with a retinal implant

**DOI:** 10.1186/1471-2202-15-S1-P95

**Published:** 2014-07-21

**Authors:** Kerry Halupka, Mohit Shivdasani, Shaun L Cloherty, David B Grayden, Anthony N Burkitt, Hamish Meffin

**Affiliations:** 1 Neural Engineering Laboratory, Dept. of Electrical & Electronic Engineering, University of Melbourne, Parkville, Australia; 2 Centre for Neural Engineering Laboratory, University of Melbourne, Parkville, Australia; 3 National ICT Australia, Victoria Research Lab, University of Melbourne, Parkville, Australia; 4 Bionics Institute, 384-388 Albert St, East Melbourne, Australia; 5 National Vision Research Institute, Australian College of Optometry, Carlton, Australia; 6 Dept. Optometry and Vision Sciences, University of Melbourne, Parkville, Australia

## 

Retinal implants for restoring partial vision to people with degenerative photoreceptor diseases consist of an array of electrodes implanted on the retina which can be driven according to images captured by a camera via stimulation electronics. In the past two decades this technology has progressed from prototypes, through clinical trials, through to the first commercial devices available in the U.S. last year (2013). Currently, the functional benefit to patients is largely as an aid to mobility and navigation. Improved outcomes, for example to the ability to perform useful object recognition, will likely require higher density electrode arrays. There is also likely to be benefit from attaining greater spatial control of electrically evoked neural activity by choosing appropriate stimulation parameters. As a first step towards this goal we investigated the ability of simple linear-nonlinear models to capture the response of visual cortical neurons driven by a retinal implant.

Using an electrode array containing 21 platinum electrodes in the suprachoroidal space of the retina in an anaesthetized cat, spatially white and temporally sparse electrical pulses were delivered to the retina simultaneously across several electrodes. Each pulse was either an anodic-first or cathodic-first charge-balanced biphasic current pulse. Multi-unit spiking responses were recorded using 6x10 arrays (Blackrock Microsystems, UT) in the primary visual cortex.

We formulated a model to predict spiking responses observed in the cortex in response to electrical stimulation of the retina. The pathway between the retina and the cortex was modelled as a set of spatial linear filters, each followed by a static nonlinearity. To encapsulate the effects of both anodic-first and cathodic-first current pulses, two different linear filters (one for anodic-first and one for cathodic-first stimuli) and their associated nonlinearities acted on the input separately. The output of these two nonlinearities was combined to produce an estimate of the spike rate at a given site in the cortex. The parameters of the model were optimized with a non-linear fitting algorithm. We used 83% of the available experimental data to fit the model parameters. After fitting the model parameters, the model accurately predicted the pulse-by-pulse spike count for the remaining 17% of the data (r=0.83).

The spatial linear filter can be considered to be the weighting matrix for the effect of the stimulating electrodes on a cortical site. We anticipate that the model may provide a basis for devising ideal stimulation strategies for shaping the activity in the cortex.

